# Supplementary figures and images for: Expanding the DNA editing toolbox: Novel lambda integrase variants targeting microalgal and human genome sequences
Source: PLoS One. 2024 Feb 13;19(2):e0292479. doi: 10.1371/journal.pone.0292479 (PMC10863862; doi:10.1371/journal.pone.0292479)

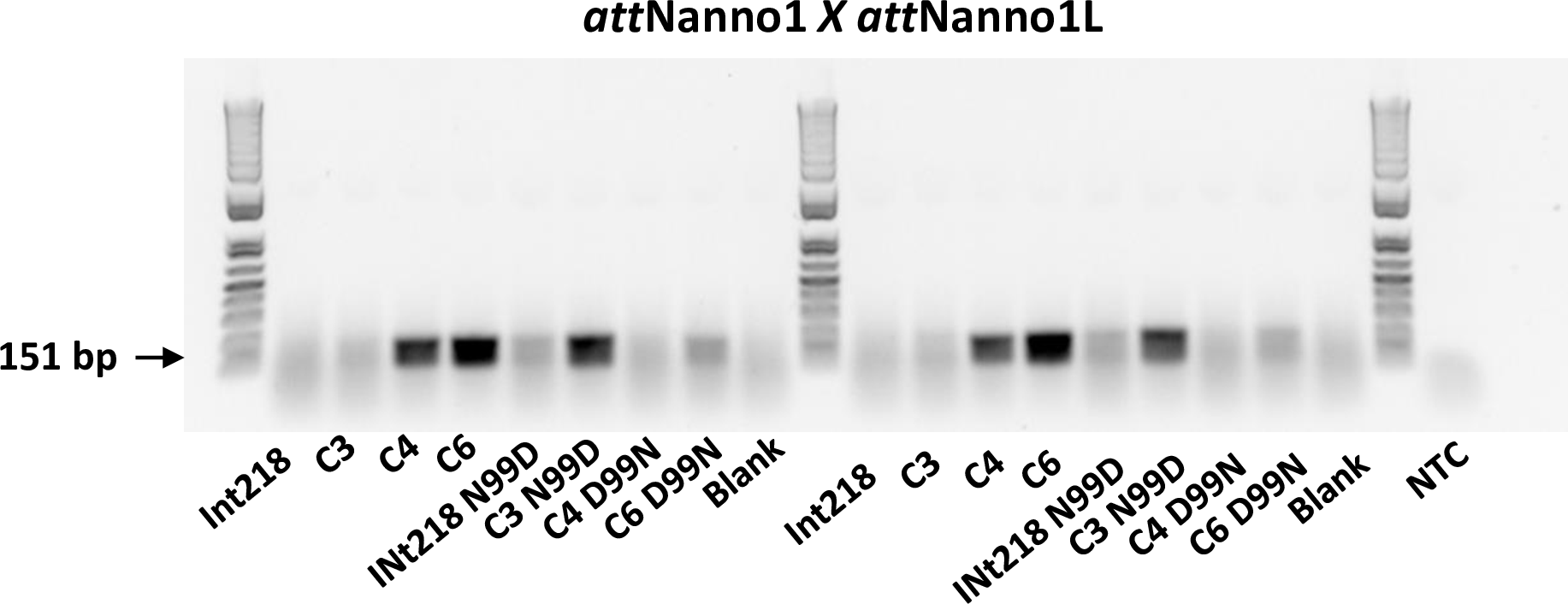

Supplement: S1 Fig — The indicated integrases were made using in vitro translation and incubated with attNanno1 X attNanno1L plasmid substrate. Scoring of recombination post incubation was carried out by end-point PCR using the primer 3 + 4 pair. Recombination measured by these primers yields a 151 bp band (arrowed). (TIF) [file pone.0292479.s001.tif]

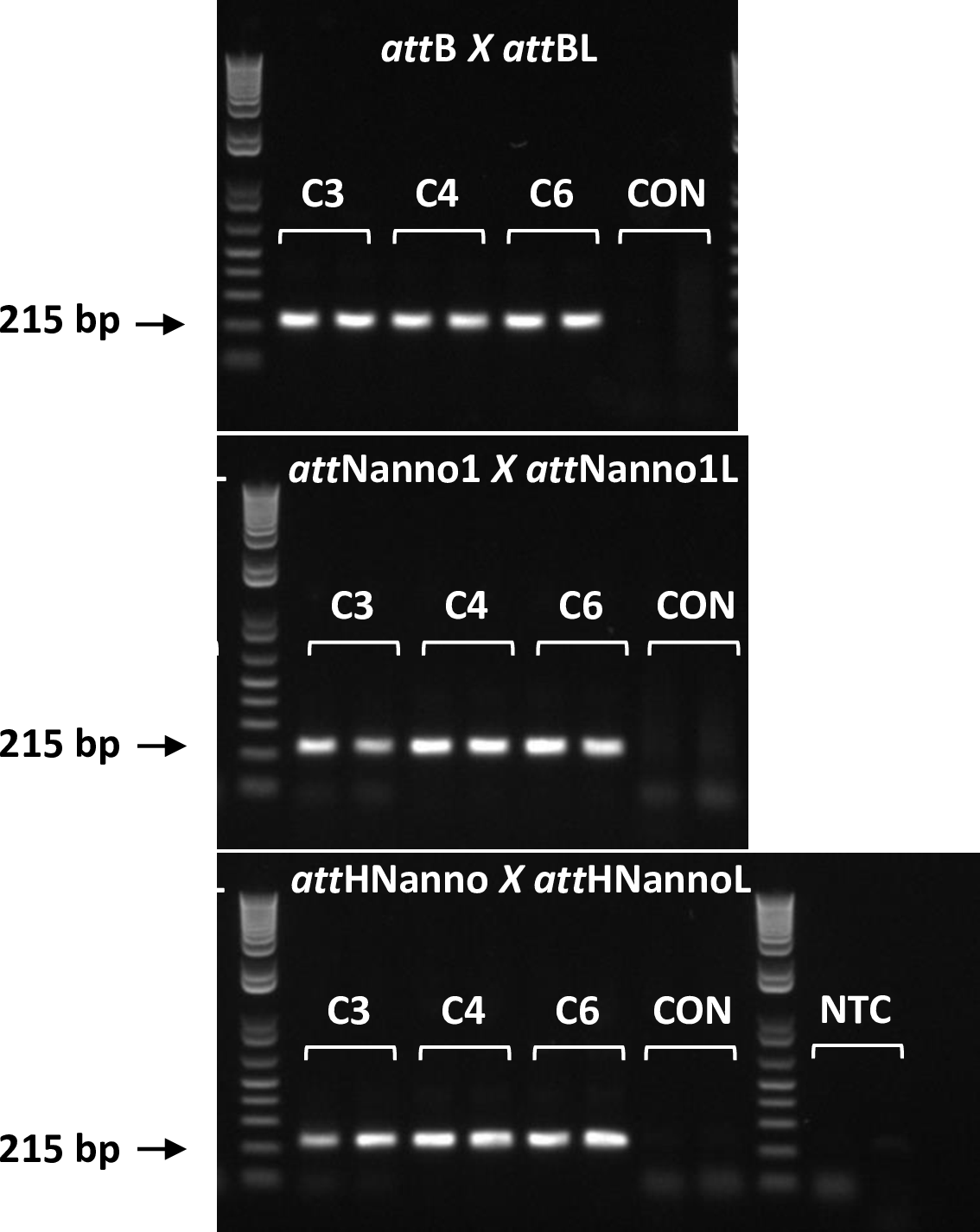

Supplement: S2 Fig — Arrow indicates size of correct band (215 bp) indicating recombination event. CON: no enzyme in recombination reaction. NTC: no template control. (TIF) [file pone.0292479.s002.tif]

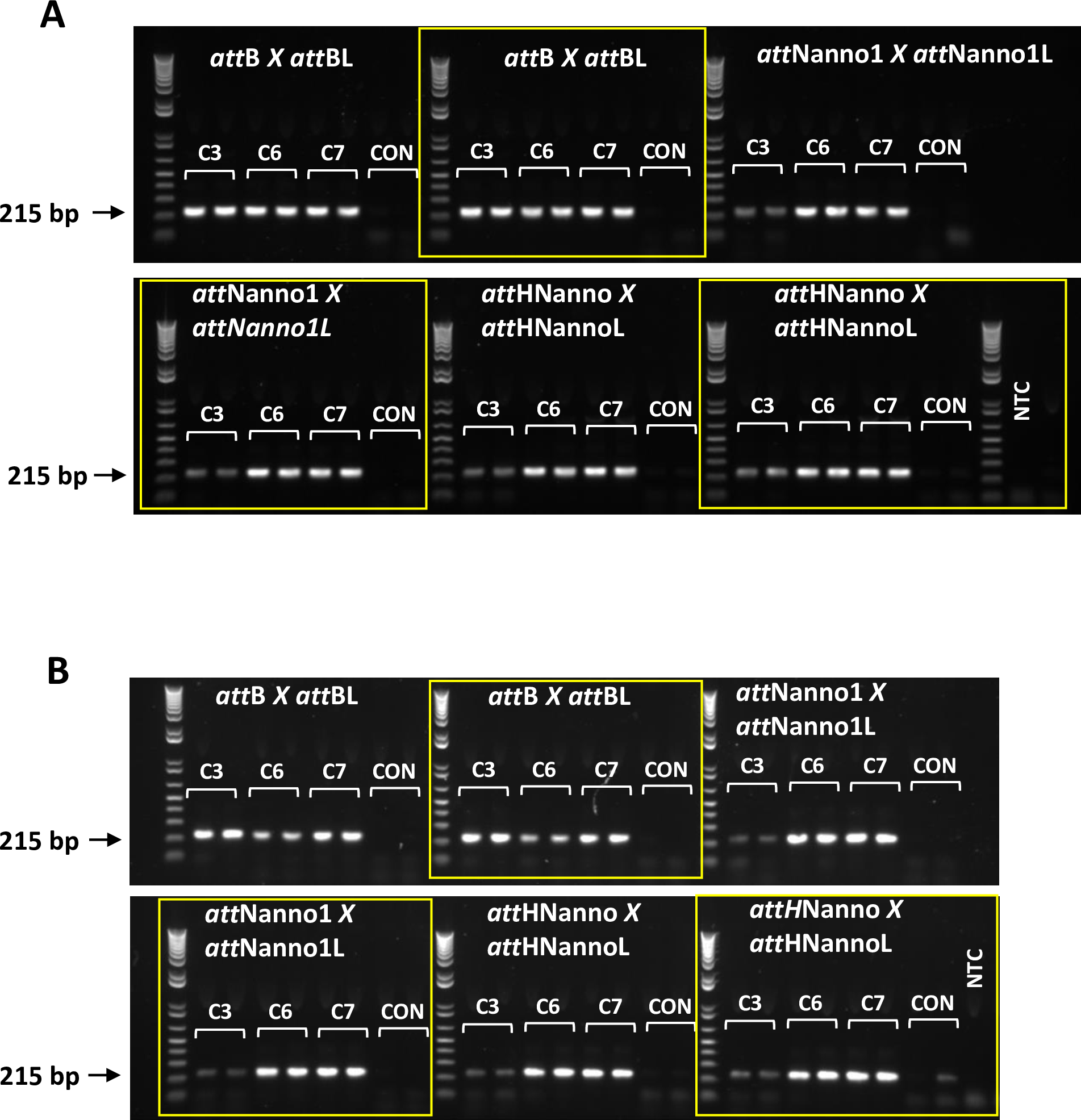

Supplement: S3 Fig — (A) Purified integrases (C3, C6, C7) were incubated with indicated plasmid DNA substrates and activity determined by real-time PCR using the primer 5 + 6 pair. Reactions comprised 17nM integrase and 1.3nM respective DNA substrate. PCR products subsequently resolved on gels shown. Yellow boxed lanes denote repeat experiment. Arrows show position of expected band indicating recombination. (B) As in (A), using 10-fold less enzyme and DNA substrate. (TIF) [file pone.0292479.s003.tif]

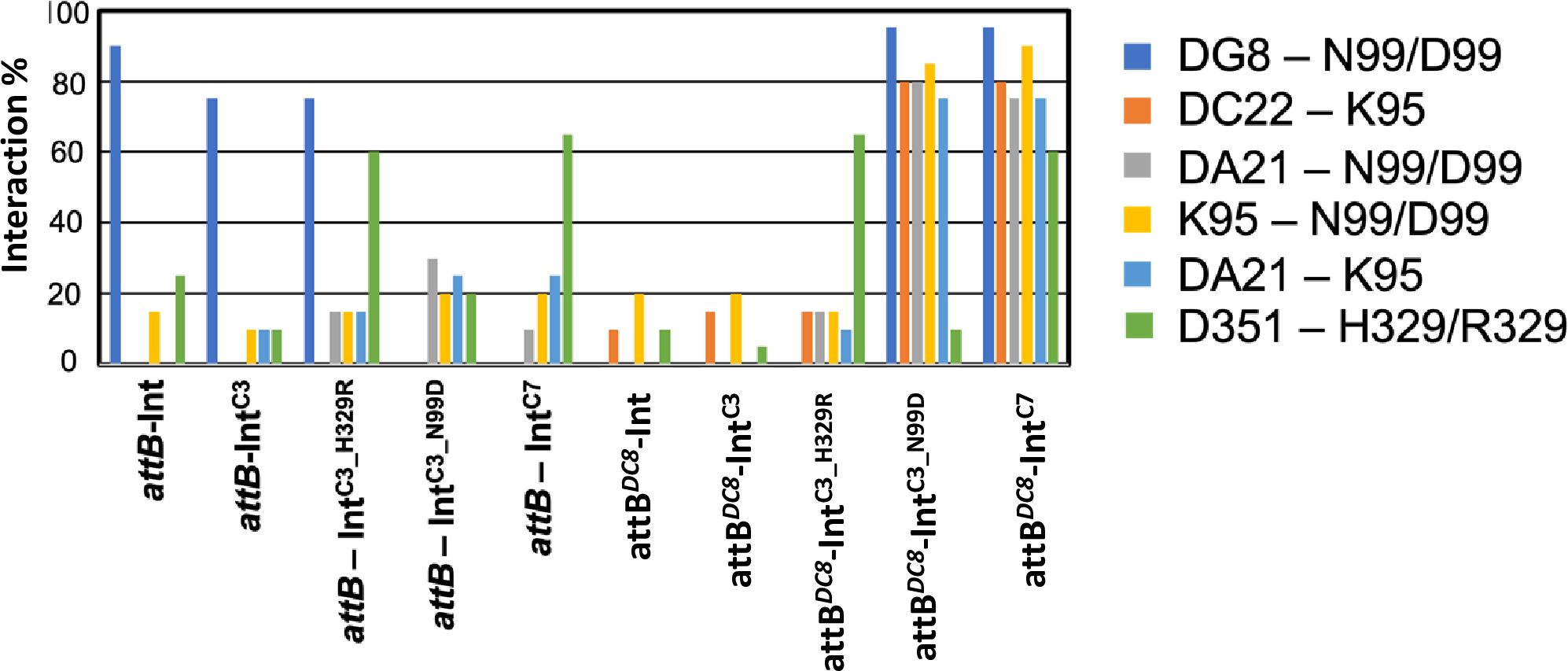

Supplement: S4 Fig — (TIF) [file pone.0292479.s004.tif]

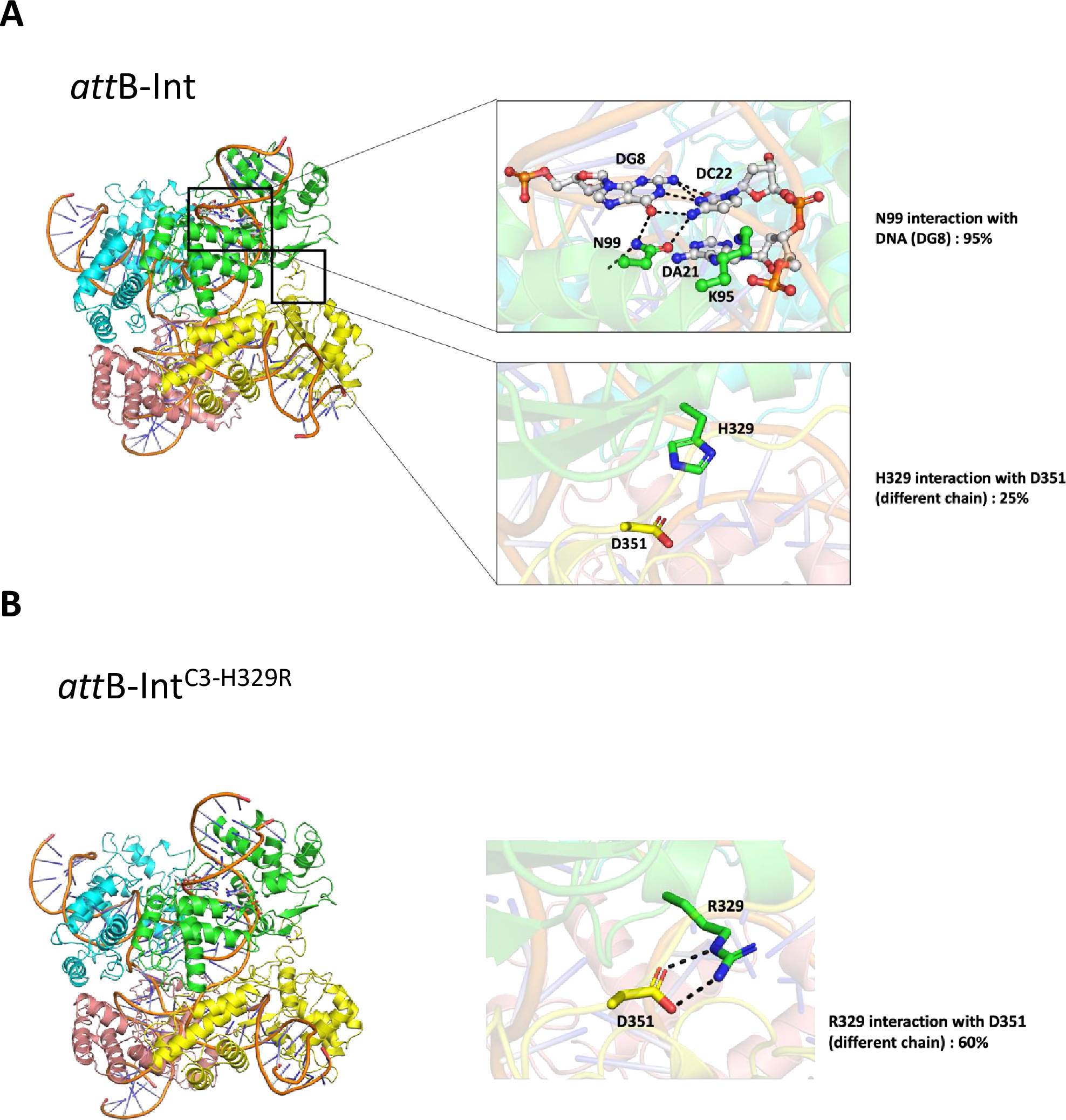

Supplement: S5 Fig — Tetrameric structure of Int shown on left with individual chains coloured differently. Boxed regions are expanded in the images on the right to show interactions with attB or the variant attBDC8 and intra-chain reactions. (A) attB-Int (B) attB-IntC3-H329R. (TIF) [file pone.0292479.s005.tif]

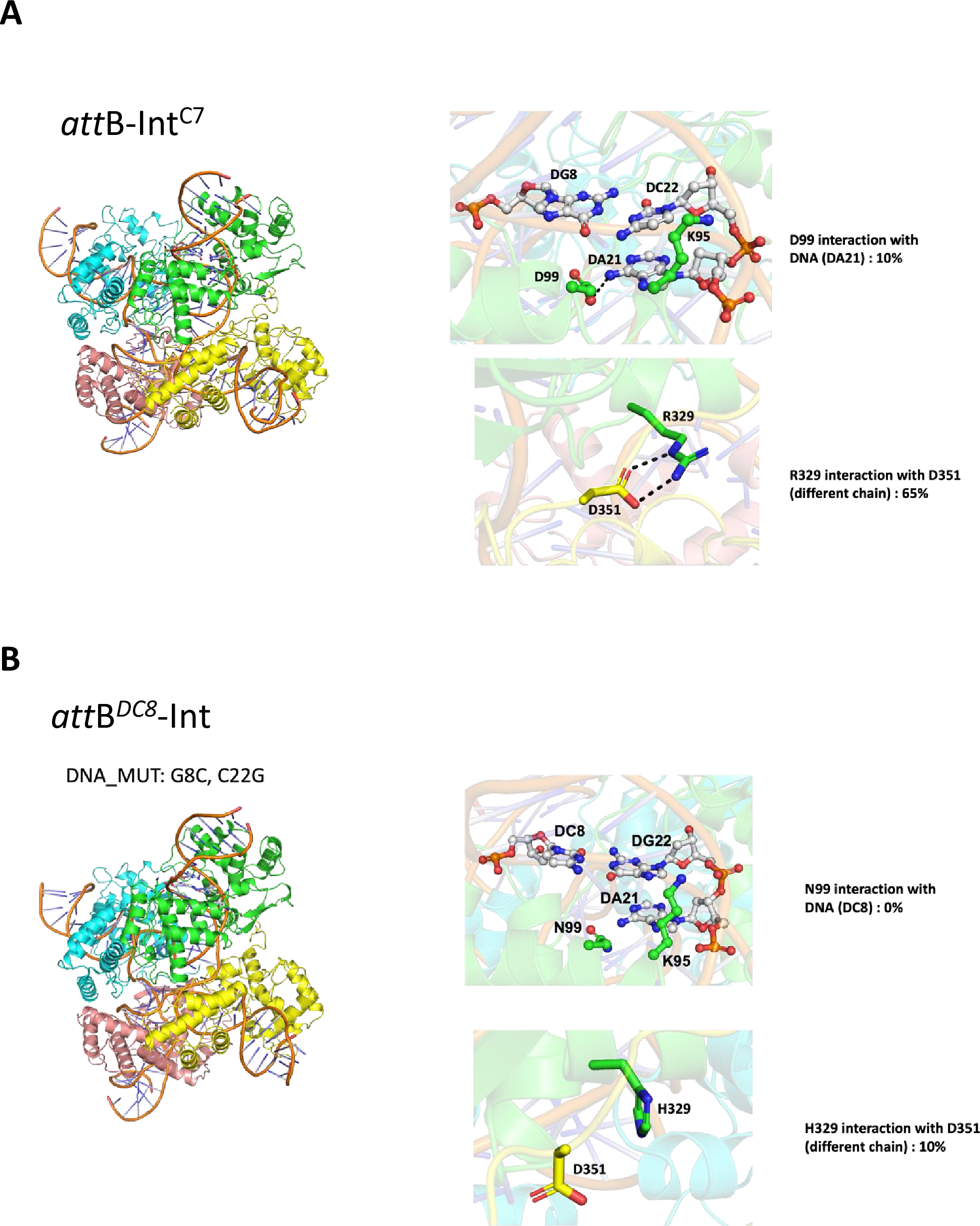

Supplement: S6 Fig — Tetrameric structure of Int shown on left with individual chains coloured differently. Boxed regions are expanded in the images on the right to show interactions with attB or the variant attBDC8 and intra-chain reactions. (A) attB-IntC7 (B) attBDC8-Int, DNA_MUT: G8C, C22G. (TIF) [file pone.0292479.s006.tif]

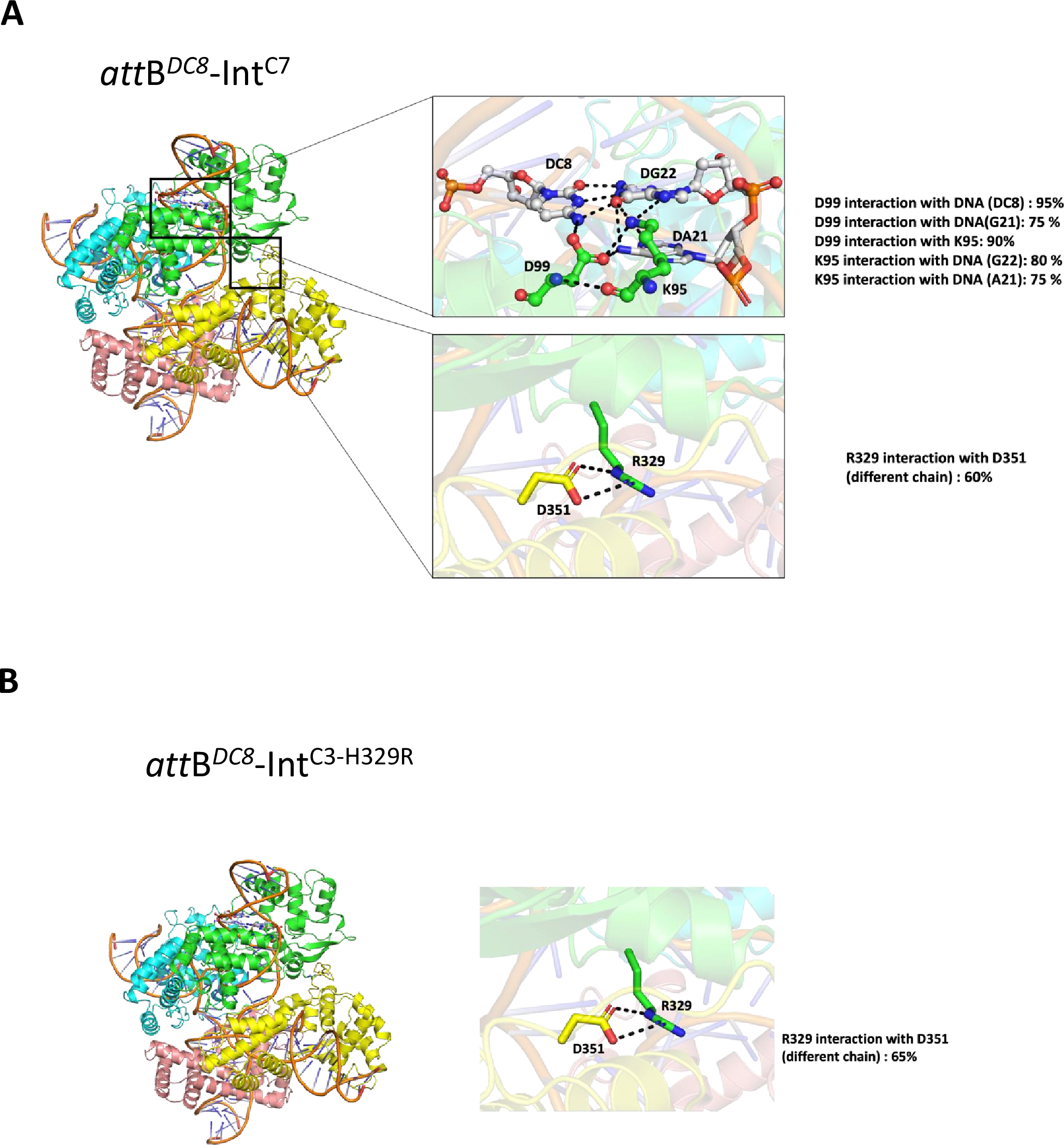

Supplement: S7 Fig — Tetrameric structure of Int shown on left with individual chains coloured differently. Boxed regions are expanded in the images on the right to show interactions with attB or the variant attBDC8 and intra-chain reactions. (A) attBDC8-IntC7 (B) attBDC8-IntC3-H329R. (TIF) [file pone.0292479.s007.tif]

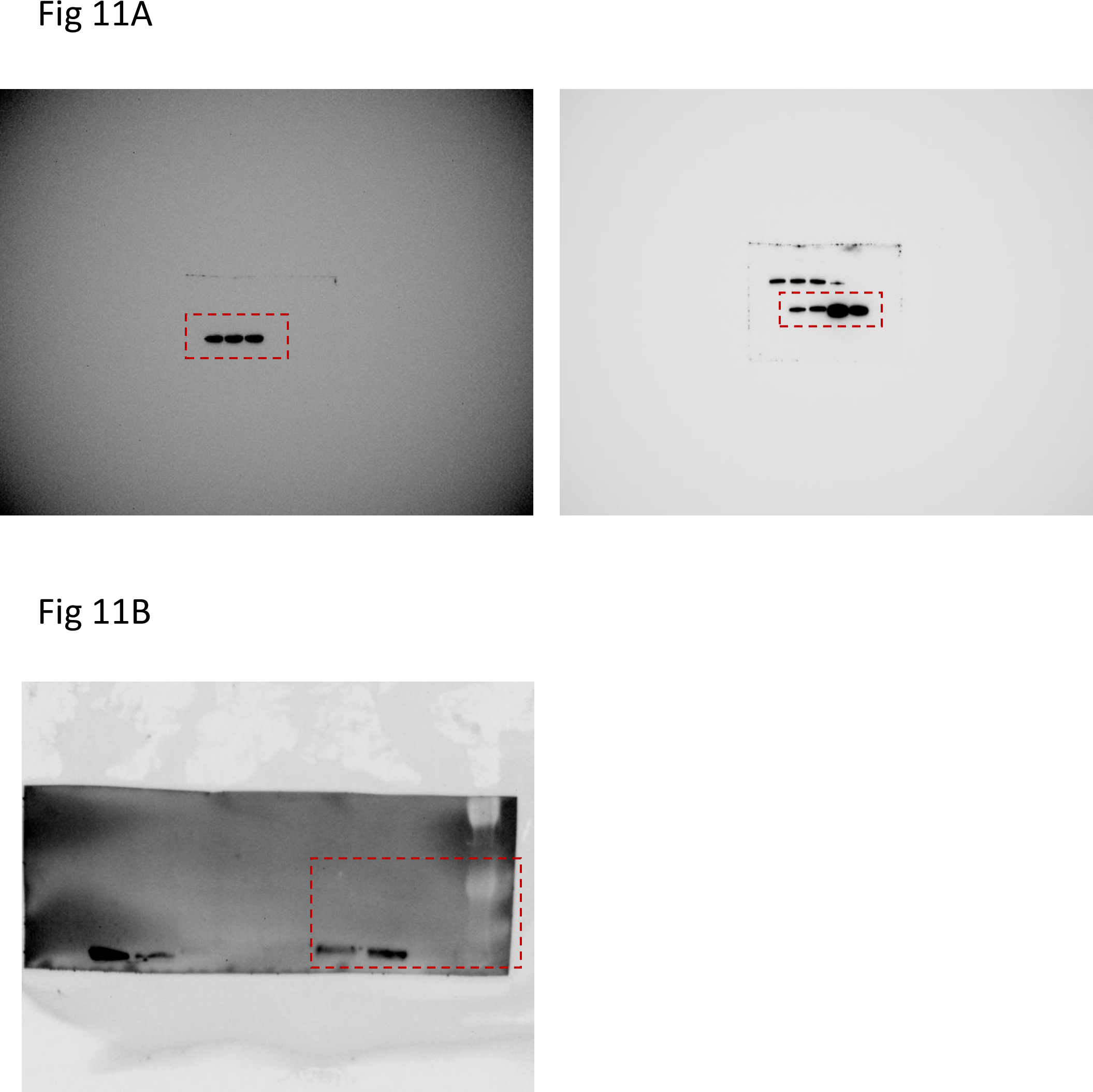

Supplement: S8 Fig — (TIF) [file pone.0292479.s008.tif]
