# Supplementary material for: Expanding the DNA editing toolbox: Novel lambda integrase variants targeting microalgal and human genome sequences
Source: PLoS One. 2024 Feb 13;19(2):e0292479. doi: 10.1371/journal.pone.0292479 (PMC10863862; doi:10.1371/journal.pone.0292479)
Supplement: S1 Table — (DOCX) [file pone.0292479.s009.docx]

| **#** | **Primer** | **Sequence (5’-3’)** |
| --- | --- | --- |
| **1** | jw278temF | AGCGTGACACCACGATGCCTGCAGCAATGGC |
| **2** | pINT-qPCR1 | GTGCCTCACTGATTAAGCATTGGCCATG |
| **3** | INVPCRF | GCTTCGATGTGACGGTTACTCGT |
| **4** | INVPCRR | GATCCGGACAGAACGTTGTTTTCAG |
| **5** | INVPCR-R2 | CGAGGACCTGCTGACGTGTCA |
| **6** | INVPCR-F2 | CGATGTGACGGTTACTCGTCGA |
| **7** | pINT-qPCR2 | GACTGATAGTGACCTGTTCGTTGCAAC |
| **8** | petR | CGGATATAGTTCCTCCTTTCAGCA |
| **9** | petF2 | CATCGGTGATGTCGGCGAT |
| **10** | RJ_gnmc_pri_rev | TTTAGTCTCCAAACTAGGCATCG |
| **11** | RJ_gnmc_nes_rev | CTTGCTGAATGAGGCTCCAACAC |
| **12** | 200 puro rev 24 | CACCGTGGGCTTGTACTCGGTC |
| **13** | 102 puro rev 102 | GCTCGTAGAAGGGGAGGTTG |
| **14** | 202 Puc ori_fwd_2 | GATAAGTCGTGTCTTACCGGGTTG |
| **15** | 21 PTZUE_attBhygro_fwd_1-seq | CACAGGAAACAGCTATGACCATG |
| **16** | LJ_gnmc_nes_fwd | CAAGAATGGAAGATTGACAGCCAG |
| **17** | LJ_gnmc_pri_fwd | ATGTGTTGGGAGTGAAGTTAGTAAGC |
| **18** | tem-INTinf-ndeF | AAGGAGATATACATATGGGAAGAAGGCGAAGTCATGA |
| **19** | tem-INTinf-ecoR | CCCCCACTTTGAATTCTTATTTGATTTCAATTTTGTCCCACTC |
| **20** | INF-INT-nde1F | AAGGAGATATACATATGGGAAGAAGGCGAAGTCATGAGCGC |
| **21** | INF-INT-HA-ecoR1R | GACGGAGCTCGAATTCTCATTAAGCGTAATCTGGAACATCGTATGG  GTATTTGATTTCAATTTTGTCCCACTCCCTGCC |
